# A modeling workflow that balances automation and human intervention to inform invasive plant management decisions at multiple spatial scales

**DOI:** 10.1371/journal.pone.0229253

**Published:** 2020-03-09

**Authors:** Nicholas E. Young, Catherine S. Jarnevich, Helen R. Sofaer, Ian Pearse, Julia Sullivan, Peder Engelstad, Thomas J. Stohlgren

**Affiliations:** 1 Natural Resource Ecology Laboratory, Colorado State University, Fort Collins, Colorado, United States of America; 2 U.S. Geological Survey Fort Collins Science Center, Fort Collins, Colorado, United States of America; 3 U.S. Geological Survey Pacific Island Ecosystems Research Center, Honolulu, Hawaii, United States of America; University of Molise, Isernia, ITALY

## Abstract

Predictions of habitat suitability for invasive plant species can guide risk assessments at regional and national scales and inform early detection and rapid-response strategies at local scales. We present a general approach to invasive species modeling and mapping that meets objectives at multiple scales. Our methodology is designed to balance trade-offs between developing highly customized models for few species versus fitting non-specific and generic models for numerous species. We developed a national library of environmental variables known to physiologically limit plant distributions and relied on human input based on natural history knowledge to further narrow the variable set for each species before developing habitat suitability models. To ensure efficiency, we used largely automated modeling approaches and human input only at key junctures. We explore and present uncertainty by using two alternative sources of background samples, including five statistical algorithms, and constructing model ensembles. We demonstrate the use and efficiency of the Software for Assisted Habitat Modeling [SAHM 2.1.2], a package in VisTrails, which performs the majority of the modeling analyses. Our workflow includes solicitation of expert feedback on model outputs such as spatial prediction results and variable response curves, and iterative improvement based on new data availability and directed field validation of initial model results. We highlight the utility of the models for decision-making at regional and local scales with case studies of two plant species that invade natural areas: fountain grass (*Pennisetum setaceum*) and goutweed (*Aegopodium podagraria*). By balancing model automation with human intervention, we can efficiently provide land managers with mapped predicted distributions for multiple invasive species to inform decisions across spatial scales.

## Introduction

The ongoing global spread of harmful exotic plant species is increasing their occupancy and abundance, posing challenges for natural areas, where successful management of natural resources may depend on prevention, containment, and control of invaders [1–3]. Knowledge of where invasive plants occur is a pre-requisite for efficient mitigation of their impact and minimization of their spread [4]. At broad geographic scales, such as across biomes, watch lists and risk assessments rely on information about the current distribution of invasive species across geographic and environmental conditions [5,6]. At local scales, such as protected areas or U.S. counties, search efforts to find new populations of invasive species and to target management actions are guided by maps of invasive plant species occurrence and modeled habitat suitability [7–9].

Species distribution models (SDMs) are commonly used to estimate potential distributions of invasive species and to assess what areas contain suitable habitat. Correlative SDMs estimate the occurrence of a species based on habitat attributes such as climate, soils, and land use [10]. These models are then spatially projected to represent the modeled species’ potential habitat and provide an objective tool to understand the distribution of invasive species. However, the credibility of different SDMs varies widely based on the quality of information used in making models, choices made in the modeling process, and to what degree natural history information and expert opinion factor into the modeling process [11,12]. Making the most credible SDM takes considerable effort and often requires collecting information that is not readily available (e.g., on species absence and detection probability), while a fully automated process using a standard set of existing information may produce many models quickly but has less credibility. Iterative modeling, the incremental improvement of SDMs based on added information and modified modeling processes over time, provides a way to minimize the tradeoffs between quality and quantity of models created and to objectively demonstrate improvement of models with the use of additional information, including new data, expert review, and field validation [13–15].

The intended use of habitat suitability model results is a critical consideration when developing SDM methodologies [16]. An automated methodology requiring little human intervention in the modeling process can quickly produce models for a large number of species. Such models can provide broad biogeographical perspectives and be informative for global and continental biodiversity assessments, but careful methodological consideration is always necessary and guidelines have been developed for these contexts [17]. In contrast, tailoring the model and input data, including collecting higher quality data than may readily exist, is appropriate for high-stakes decisions like those involving regulatory decisions [18]. Invasive species often sit at an intermediate point in terms of data availability, stakes of the decisions, and resources available for modeling and management. The sheer number of established and potential exotic plant species for which SDMs might provide critical distributional information remains a major challenge [19,20], and at the same time, credibility for species-specific management responses is often needed. Striking a balance among these aspects of SDMs can be a challenge, especially when multiple species and multiple spatial scales are of interest.

Management of invasive plant species occurs at both broad and local scales and often requires combining multiple analyses and reviewing multiple maps (Table 1). National-extent maps are often too coarse in resolution (e.g., 1 km) for decisions within a management unit, while fine resolution, small-extent (local) maps only include a tiny portion of the landscape, making regional decisions and cross-agency coordination difficult. A small number of invasive species cause a majority of the impacts to local biodiversity, ecosystem functioning, and human well-being [21], but across large or diverse geographic areas, there may be many species of management interest. Moving forward, the scope of species distribution models needs to be inclusive of large geographic regions, while the geographic precision of models needs to be fine-scale enough to make predictions within an individual management unit. Developing a set of input data covering a large geographic extent at a fine resolution for use in modeling the broad set of invasive species of management interest could make a single set of models useful for local targeting in the invaded range and regional or national risk assessment beyond the current range boundaries.

**Table 1:** Invasive species distribution model interpretation scales (national, regional, local) and their audience, use and select examples from the literature.

Scale	Extent Example	Audience	Primary Use
**National**	Contiguous U.S.	Federal land management agencies, National policy actors, Federal plant and animal health protection organizations	Understand the potential scope of the problem [22,23]. Characterize the threat. Resource planning and allocation among regions [15,24]. Restrictions on international trade. Guide higher-resolution models at smaller extents [25]
**Regional**	Exotic Plant Management Team (EPMT) regions, States, Ecological zones, Watershed	Regional coordinators (e.g., Great Lakes Early Detection Network), District Managers, State weed agencies, Conservation organizations	Coordinate invasive species surveys [26]. Inform local watch lists, assessing & communicating regional risks [24,27–29]. Invasion prevention and management [26]
**Local**	National Park, Reserve, National Forest, County, Disturbance area (e.g., fire)	Land managers, Local government agencies	Early Detection and Rapid-Response [9]. Monitoring invasion spread, developing containment strategies [8,24]. Inventory of the scope of the invasion at local scale [25]

**Fig 1:**
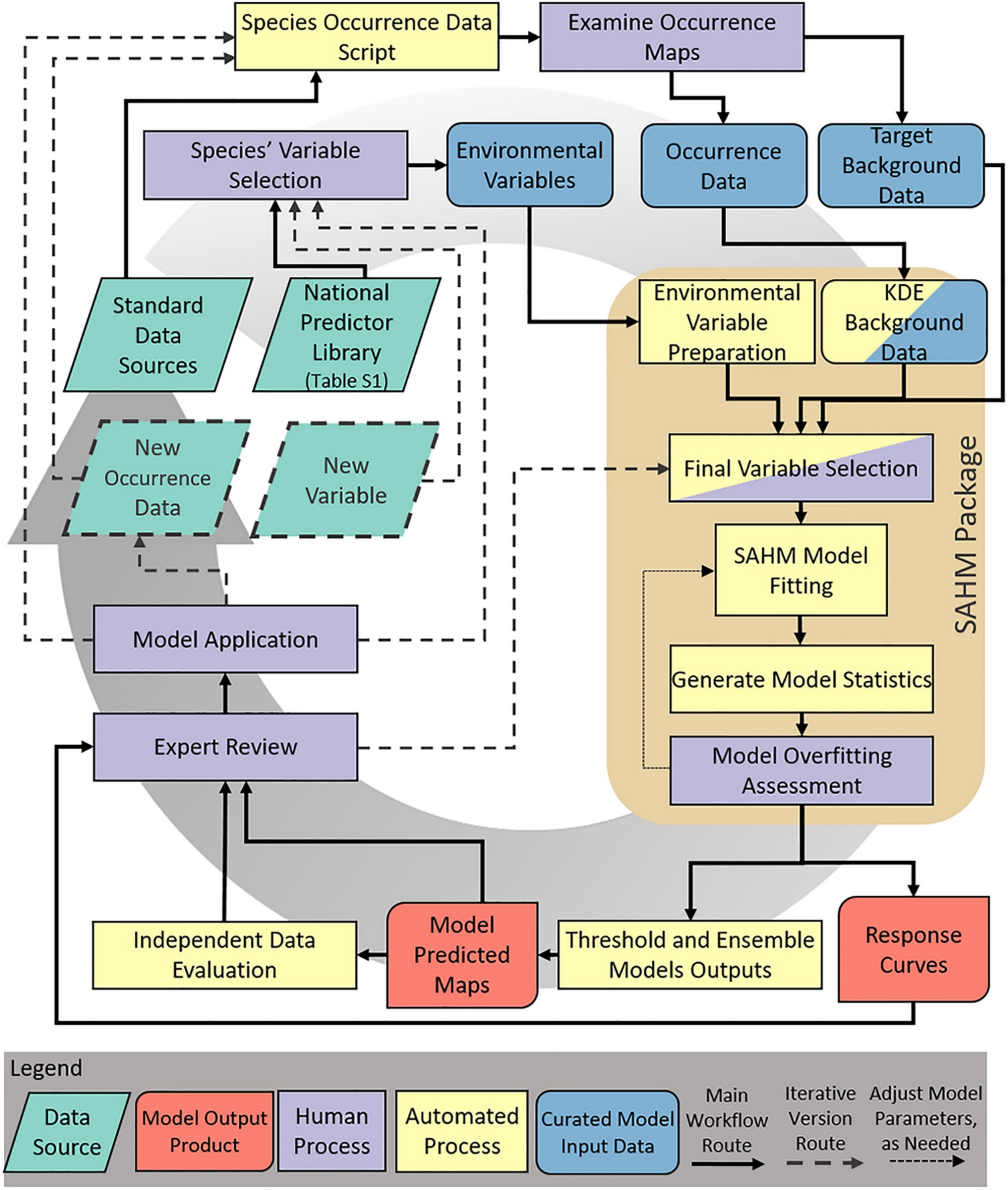
Workflow of the modeling framework showing data sources, model input data, automated and human processes, model output products and the paths for model iterations.

Here, we outline a robust and efficient methodology for the initial development of multiple, credible SDMs of invasive plant species. Our objective is to establish a modeling protocol that leverages existing data and balances automation with human intervention to perform model analyses for several species using a curated suite of contiguous U.S. variables at 90 m resolution that could be useful at national, regional and local scales. The methods also utilize improved computing power to create models with a large extent and fine grain size to deliver results useful at multiple spatial extents (Fig 1). We use two invasive plant species to illustrate our model development process, model validation using independent data, and model utility for management: fountain grass (*Pennisetum setaceum* [Forssk.] Chiov.), a broadly-established invader in the U.S. since the 1940s, and goutweed (*Aegopodium podagraria* L.), an invader in the U.S. since the 1860s, but with a more limited distribution.

## Methods

### Occurrence and background data

We downloaded occurrence data for each species from multiple databases. We created a species occurrence data script in the statistical software program R [30] to create a consistent occurrence data acquisition workflow for the modeled species (‘Species Occurrence Data Script’, Fig 1, https://doi.org/10.5281/zenodo.3581395. First, all known synonyms and U.S. Department of Agriculture (USDA) Plants Database [31] acronyms were collected (excluding subspecies, variants, and hybrids) using the Integrated Taxonomic Information System (ITIS; www.itis.gov) as an authoritative taxonomy in the R library ‘taxize’ [32]. Next, occurrence data repositories were queried from online sources: the Global Biodiversity Information Facility (GBIF [33]), Biodiversity Information Serving Our Nation (BISON [34]), and the Early Detection & Distribution Mapping System (EDDMapS [35]). Records from the Bureau of Land Management’s (BLM) and the National Park Service (NPS) National Invasive Species Information Management System databases and data from the BLM Assessment Inventory and Monitoring program were added to these results. We filtered the aggregated data by observation type (observation or specimen only), observation date (1980 to present), and coordinate uncertainty (≤ 30 m). We removed any records with coordinates corresponding to state or country centroids or other identifiable geographic and taxonomic errors. We also checked the entire dataset for duplicate records. We obtained data for fountain grass (accessed 24 July 2018) and goutweed (accessed 29 April 2019) [33–35]. Once downloaded and filtered, the data were mapped and visually checked for accuracy (‘Examine Occurrence Maps’, Fig 1). We had 3,422 unique fountain grass locations and 1,168 unique goutweed locations for model development that resulted from the data download script.

We used a background sample approach to develop SDMs for each species since absence data are rare and can be unreliable for invaders that are still expanding [36]. We developed models using two background method generation approaches since the choice of background method, including location extent and placement, can strongly affect model results [37]. First, we developed a continuous kernel density estimation (KDE) layer around the occurrences for each species (termed ‘KDE Background Data’, Fig 1), restricted in extent to a minimum convex polygon around occurrences that was buffered by 1/3 of the maximum difference between minimum and maximum X coordinates and minimum and maximum Y coordinates. This KDE approach results in the background sample density correlating with the occurrence point density and was proposed as an appropriate method for spreading invasive species by up-weighting locations that may be at the invasion front [36]. Our second background sampling approach was the target guild method (termed ‘Target Background Data’, Fig 1), which uses presence locations from similar species as background points [38], to mimic sampling bias in the presence data under the assumption that locations from the same data sets for similar species would have the same sampling biases. To develop the background samples for this ‘target’ model methodology, we obtained a list of species from the USDA Plants Database designated as introduced across the entirety of the continental United States. We then obtained aggregated data following the protocol above for all introduced species. We restricted the extent from which we selected target background locations to a binary 99% isopleth KDE based on the presence locations to ensure we were limiting them to accessible areas [39]. To model a focal invader, we subset the introduced species occurrences based on the life form of the species of interest (e.g., grass for fountain grass and forb or herb for goutweed) as defined by USDA Plants Database. These occurrences, filtered by lifeform, became our target background data and were additionally checked for accuracy by mapping their distribution.

### Environmental variables

We compiled a national library of potential environmental variables to consider for the distribution modeling. We only considered variables available throughout the contiguous U.S (‘National Predictor Library’ Fig 1). These variables were compiled based on consideration of what environmental factors may limit plant species’ distributions and we selected environmental variables that captured attributes such as water balance and temperature extremes known to physiologically limit plant distributions [40]. We included the most up-to-date representation of climatic, topographic, soil, land use, and anthropogenic factors that have coverage across the contiguous U.S. at the highest spatial resolution available (Table A in S1 File). The curation of this library of variables was intentional and rigorous, and included custom derived metrics and widely-used canned datasets such as Bioclim [41]. A total of 60 environmental variables were compiled and were considered for model development (Table A in S1 File). All environmental variables were processed to the Alber’s Equal Area coordinate reference system, continental U.S. extent, cell size (90 m) using the nearest neighbor method of resampling and alignment using the *PARC* (Project, Aggregate, Resample, Clip) module in the Software for Assisted Habitat Modeling (SAHM, [42]) (‘Environmental Variable Preparation’, Fig 1).

A critical aspect of our modeling workflow was the species-specific selection of predictor variables, which used a combination of automatic and human input processes (‘Species’ Variable Selection’ and ‘Final Variable Selection’, Fig 1). First, we conducted a literature review for each species to help inform which environmental variables from the national library we compiled limit the distribution of each species. We also drew on our own natural history knowledge of each species, and on initial conversations with management practitioners in the regions where these species are impacting ecosystems. We retained predictors for modeling from the national library based on this species-specific information and by removing one of any pair of variables that had a maximum of the Pearson, Spearman, or Kendall correlation coefficient |r|> 0.7 [43] to reduce issues of collinearity. This analysis was accomplished using the *CovariateCorrelationAndSelection* module in SAHM (‘Final Variable Selection’, Fig 1).

### Habitat suitability modeling

We implemented multiple model algorithms for each species since statistical model choice is the greatest source of quantifiable uncertainty in species distribution modeling [44,45], allowing us to evaluate and reduce the potential biasing effects of algorithm choice on our results. These included Maxent (v 3.4.1), Boosted Regression Trees (BRT), Random Forest (RF), Generalized Linear Model (GLM) and Multivariate Adaptive Regression Splines (MARS). We used SAHM [42] within the VisTrails software framework [46] to fit models to each combination of algorithm and background method (‘SAHM Model Fitting’, Fig 1). SAHM provides an established, streamlined and replicable approach to preprocess model inputs, run multiple algorithms and compare results. Three of the five models have an internal variable selection process; BRT used bag fraction = 0.5; GLM used a bidirectional stepwise procedure using Akaike’s Information Criterion (AIC), considering all interactions and squared terms; and MARS used Mars Degree (Friedman’s μ) = 1 and GVL penalty = 2.0. Both Maxent and RF retain all variables provided to them. We used a default set of parameters for each model algorithm, and subsequently evaluated model-specific parameter settings for any resulting model where the training and test area under the receiver operating characteristics (AUC-ROC) values based on 10 fold cross-validation had a difference > 0.05, which we defined *a priori* as a threshold to indicate overfitting along with visual inspection of response curve complexity (e.g.,[47], ‘Model Overfitting Assessment’, Fig 1). We developed 10 models for each species (five modeling algorithms each with two background sampling approaches) to estimate suitable habitat. Any model that was either still overfit or had poor assessment metrics (i.e., test AUC-ROC < 0.7 [43]) was dropped from further analysis. We produced a multivariate environmental similarity surface (MESS) for each model [36] and overlaid this surface on maps to highlight areas of extrapolation beyond the environments captured by the training data. All models were developed using the training data described above and applied to predictors for the contiguous U.S. at a spatial resolution of 90 m.

Each model was evaluated using a 10-fold cross-validation approach [48], which partitions the training data into 10 equal subsets and runs 10 model iterations where 9 subsets are used for model development (training data) and the final subset is used to test the model performance (test data). The 10 iterations are then averaged together to provide a final measure of model performance. SAHM produces several statistical measures (‘Generate Model Statistics’, Fig 1) including AUC-ROC, AUC-precision recall (AUC-PR), Kappa, True Skill Statistic (TSS), percent correctly classified (PCC), sensitivity, and specificity which we compiled for each model on the training and test data to evaluate performance. These metrics together can help evaluate the quality of a model and generally a model with a test AUC value >0.7 is considered to have good performance and can be useful [49].

### Model outputs and iteration

Each continuous model was classified to represent suitable and unsuitable habitat using the minimum predicted presence (MPP) threshold, one percentile threshold, ten percentile threshold, and the maximum sensitivity plus specificity (MSS) threshold. Each threshold falls on a spectrum from a more restrictive to more inclusive prediction of habitat suitability. Depending on the intended use of the model, one threshold may be preferred over the other or multiple thresholds may be used to present different representations of habitat suitability for an invasive species [12,50,51]. Therefore, we provide multiple threshold options in our modeling workflow that can be considered by practitioners. The MPP, one percent and ten percent thresholds are determined by the value at which the minimum, lowest one percent or lowest ten percent of the training data occurrences was classified as being in unsuitable, respectively. The MSS threshold is the value of the maximum of the true positive rate (sensitivity) plus the true negative rate (specificity). For statistical evaluations of the models, we used the value at which sensitivity equals specificity. Finally, all ten binary models for each species were combined to create a single, equal weight model ensemble map to represent the number of models predicting suitable habitat for the focal invasive species at a given threshold (‘Threshold and Ensemble Model Outputs’, Fig 1). Along with these mapped outputs, SAHM produces variable response curves showing the general relationship between each variable and suitability for the species and a measure of variable importance. We evaluated variable importance for each model based on change in the AUC statistic (ΔAUC) when values for that variable were permutated between presence and background, then ranked variables within each model relative ΔAUC.

Resource managers pointed us to an independent data set to evaluate the fountain grass models in California, CalFlora (accessed 12 December 2018 resulting in 898 cleaned occurrence records not contained in the training occurrence data set [52]). These were used as an independent validation data set for fountain grass to evaluate how many occurrences were captured at each model ensemble value for each threshold and provides a valuable resource to independently validate model performance (‘Independent Data Evaluation’, Fig 1). We did not have an independent test data set for goutweed. We also categorized the model ensemble for each species using a model assessment rubric to describe each model’s attributes related to species data, environmental predictors, modeling process, and model products using three classifications: ‘interpret with caution’, ‘acceptable’, or ‘ideal’ following Sofaer et al. [12] who defined criteria to classify each specific attribute of the model process into each of these categories. These classifications are meant to inform use, where different uses may require different levels of classification (e.g., model to guide search to improve model versus a model used for regulatory decisions).

### Case study species

We selected two invasive species of interest to managers of natural areas: fountain grass (*Pennisetum setaceum* [Forssk.] Chiov.) and goutweed (*Aegopodium podagraria* L.). Fountain grass is a perennial C4 bunch grass native to North Africa and the Middle East, but has been widely planted as an ornamental in the U.S. and has expanded into natural areas [53]. The species is most problematic in the southwest U.S. and Hawaii, where it quickly outcompetes native flora creating a monoculture that becomes a fire hazard and alters fire regimes in systems that are not historically fire-adapted [54]. Fountain grass has been established since the 1940s [55] but continues to expand into new environments where local early detection and rapid-response efforts can reduce the rate of spread, achieve local containment, or reduce impacts [56].

Goutweed (or Bishop’s weed) is a creeping, ornamental herbaceous perennial from Europe that can grow up to 1 m tall. It was reported as potentially invasive as early at 1859 [57], and is currently distributed in the northeast, northern Midwest, and northwest U.S. It reproduces vegetatively via rhizomes, and can spread aggressively once established. Where it becomes invasive, it can form dense patches that outcompete native vegetation and is difficult to remove once established [57]. Goutweed is listed as one of the most common invasive species for Pictured Rocks National Lakeshore.

### Study area

**Fig 2:**
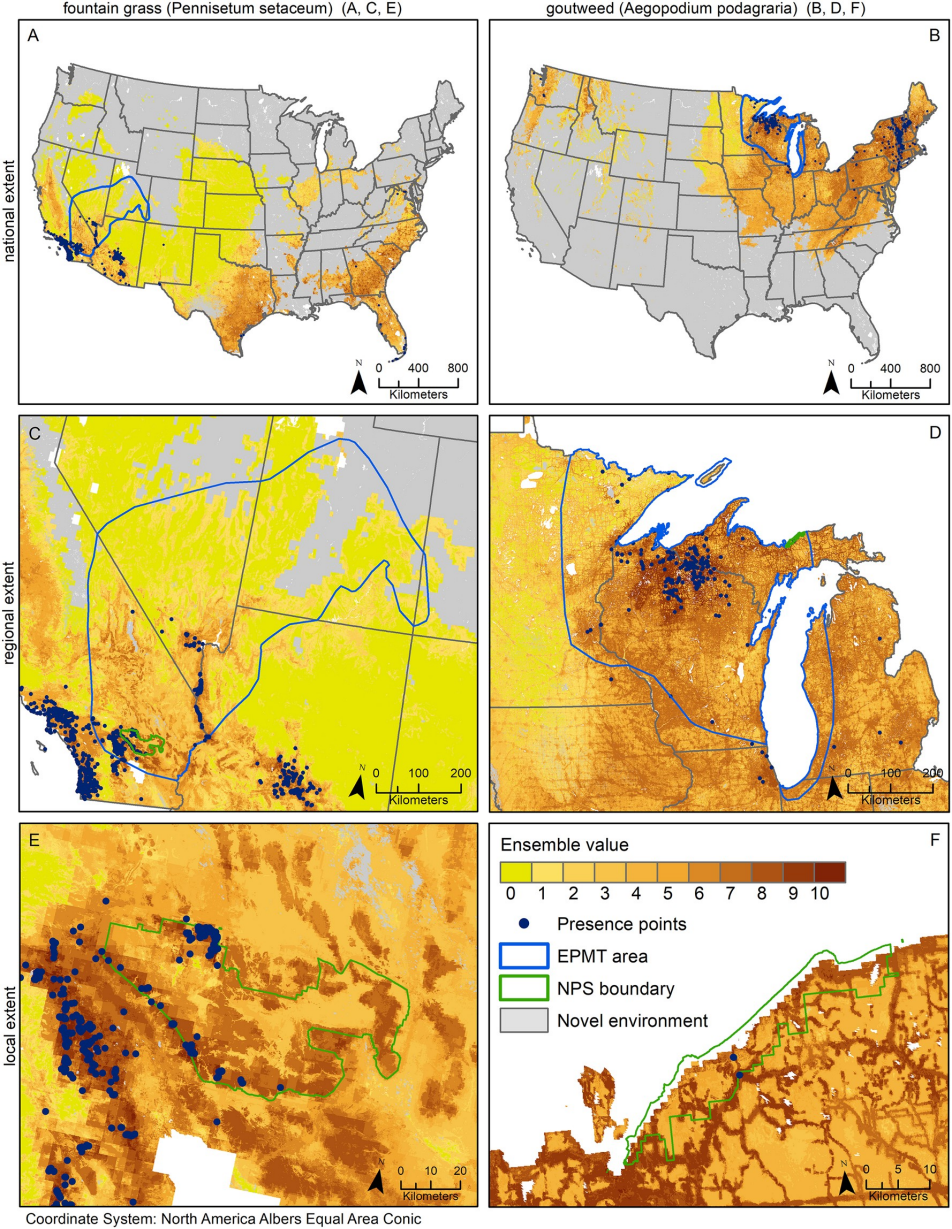
A-F: Potential habitat suitability model ensemble (maximum value of 10) using the one percentile threshold for fountain grass (*Pennisetum setaceum*) (A,C,E) and goutweed (*Aegopodium podagraria*) (B,D,F) at each extent: national extent (A, B), regional extent defined by the Exotic Plant Management Team Regions including C) Lake Mead and D) Great Lakes), and the local extent defined by E) Joshua Tree National Park and F) Pictured Rocks National Lakeshore.

We focus on three scales of invasion risk map interpretation for each species (Fig 2). At the largest extent, both species were evaluated at the contiguous U.S. At the regional scale, we used the National Park Service Exotic Plant Management Teams (EPMT) geographical regions. These regions serve as the source of invasive plant management expertise and field support to the national parks within each boundary. This may include inventory, treatment, and ecological restoration. For fountain grass, we focused on the Lake Mead region and for goutweed we focused on the Great Lakes region. Finally, at the local extent, we evaluated models for individual national park units. These units were Joshua Tree National Park and Pictured Rocks National Lakeshore for fountain grass and goutweed, respectively (Fig 2). For regional risk assessment from the perspective of the NPS EPMT, we considered the number of park units within their focal management regions containing suitable habitat for the species. We used the percentile threshold to calculate identify the park units and the area of suitable habitat within each park unit. We also conducted a nearest occurrence location analysis to provide the distance to the nearest known occurrence if a park unit was not identified to have suitable habitat.

## Results

### Model characteristics

We retained 16 variables for fountain grass model development (Table B in S1 File), although the set varied depending on background method and differences in collinearity. The human influence index and minimum winter temperature consistently fell in the top three variables ranked by percent contribution across model runs. For target background runs, spring mean potential evapotranspiration was also in the top three variable grouping. For KDE background runs, over-winter evapotranspiration completed the top three except for BRT where it ranked fourth. Fountain grass habitat suitability was associated with higher minimum winter temperatures and moderate human influence (Figure A in S1 File). It was also generally associated with higher over-winter evapotranspiration values and lower spring mean potential evapotranspiration.

The models for goutweed retained 18 environmental variables (Table B in S1 File). The most important variable for goutweed models with KDE background was the landscape condition model (ranging from 34% to 54%), with maximum mean summer temperature in the top three for three of the KDE models. For the target background runs, average annual precipitation, average minimum winter temperature and summer potential evapotranspiration were generally the three most important variables. Goutweed habitat suitability decreased with increasing landscape condition and suitability was dependent on having at least 1,500 mm of mean annual precipitation (Figure A in S1 File).

### Model performance

All model performance metrics are specific to the focal dataset, and are generally higher for lower prevalence species. Metrics provide a reliable comparison among models for a given species and dataset, while comparisons of model performance across species are confounded by variation in prevalence. Random cross-validation indicated reasonable model fit for fountain grass, with the minimum AUC and AUC-PR values across all ten models > 0.91 and > 0.79, respectively (Table C in S1 File). Threshold-dependent performance metric values (at the Sensitivity = Specificity threshold) for PCC, Kappa and True Skill Statistic values were all > 83%, > 0.62 and > 0.67, respectively. In general, BRT and RF models had the highest cross-validation statistics while GLM and MARS had the lowest. Models utilizing the KDE background method performed better than the target background models with the exception of the GLM model.

Goutweed models had cross-validation AUC values > 0.88 (Table C in S1 File). AUC-PR values varied, with the MARS KDE model having the lowest performance at 0.43 and the BRT target model having the highest at 0.75. The threshold-dependent evaluation metrics were also mixed with Kappa ranging from 0.34 (GLM KDE and MARS KDE) to 0.69 (RF target). The True Skill Statistic was more similar across models with all values > 0.64. Similar to fountain grass models, BRT and RF generally performed better than GLM and MARS. However, the target background models performed better than the KDE background models for goutweed (Table C in S1 File).

**Fig 3:**
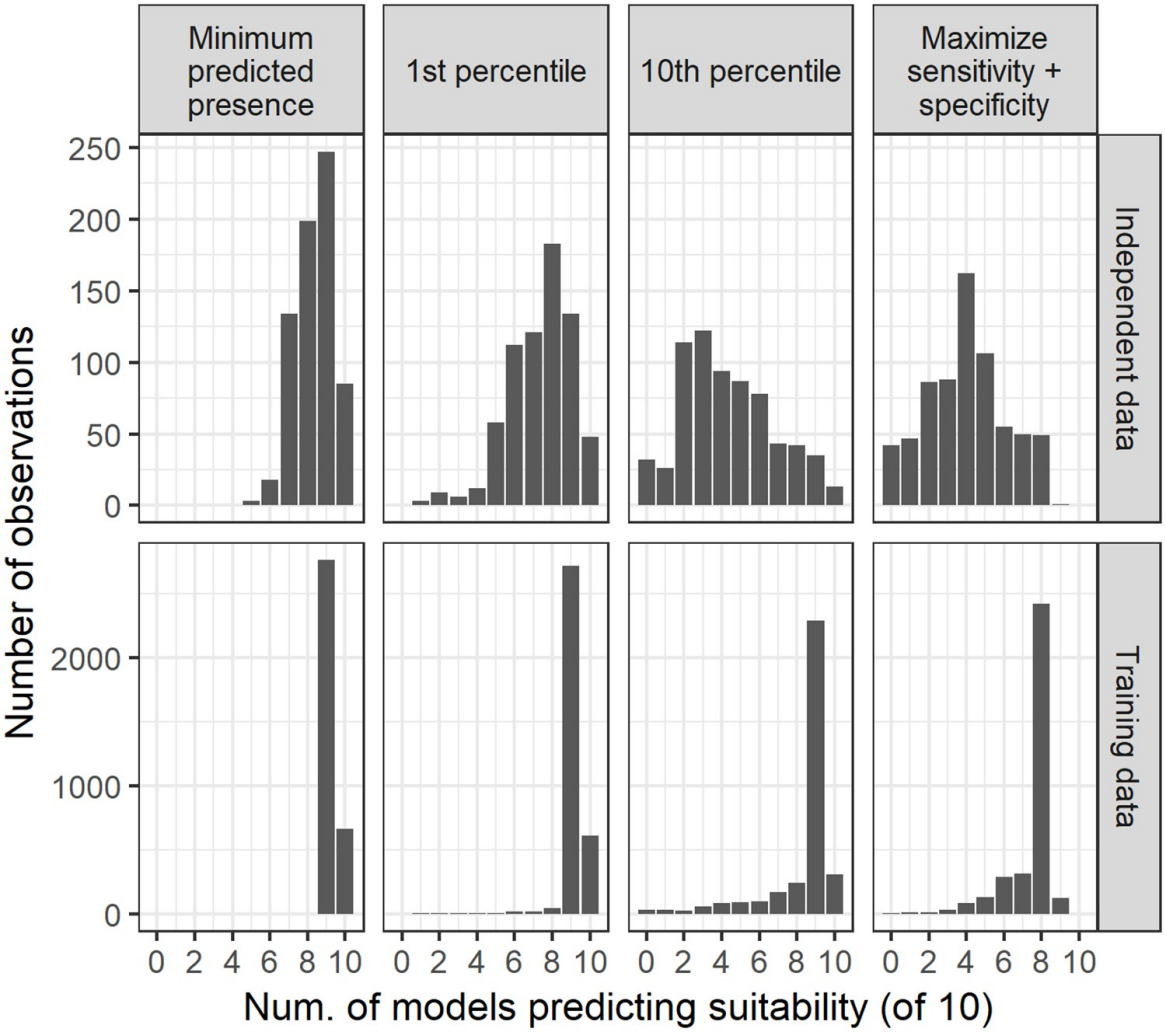
Model ensemble values associated with the independent observation data for fountain grass from CalFlora for four different threshold metrics including minimum predicted presence, one percentile, ten percentile and the maximum of sensitivity plus specificity.

The CalFlora independent dataset for fountain grass supported good performance across the threshold model ensemble (Fig 3). The majority of the occurrences were in areas where six or more models agreed across all thresholds. As expected, the most restrictive 10th percentile model ensemble (i.e., where 10% of training points are excluded from the binary definition of suitability) had the lowest sensitivity, with most of the independent records located in areas where three to six models agreed. In the inclusive MPP threshold model ensemble, all occurrences were located in areas where seven or more models agreed.

### National spatial results

The national map for fountain grass using the one percentile threshold model ensemble showed high suitability in the southwestern U.S. extending from California to Texas (Fig 2). The map also highlighted the southeast from Florida to South Carolina as potentially suitable for fountain grass. The national map for goutweed (also using the one percentile threshold) predicted relatively high suitability in the northeast and Midwest U.S. It also showed moderate suitability in the northwest where goutweed has also become a problematic invasive species. Across the U.S., there was a large amount of extrapolation in all models as indicated by the MESS maps (Fig 2). Fountain grass has areas of extrapolation in the northern third of the contiguous U.S. and portions of the southeast. Goutweed model extrapolation occurred across much of the west and the southeastern U.S.

### Regional risk assessment

The regional map of the model ensembles at the one percentile threshold reported suitable habitat in several parks not known to contain the species (Tables 2 and 3). There are 14 parks that intersect the Lake Mead EPMT region, including seven with predicted suitable habit (ranging from < 1% to 64% of the park unit) and two with known occurrences. Two of the parks that have predicted suitable habitat and no known occurrences were within 20 km of a known location. There are 12 parks occurring in the Great Lakes EPMT region, all of which are predicted to contain at least some suitable habitat for goutweed ranging from 27 to 20,373 ha, representing 9% to 98% of park units. Three of the 12 parks had occurrence records in the aggregated data used to develop the model, with an additional six parks having a location within 15 km of the park boundary.

**Table 2:** Regional analysis table for a) fountain grass (*Pennisetum setaceum*) in Lake Mead EPMT unit including the potential suitable area for the one percentile threshold, percent of park is the percent of the park area that is classified as potentially suitable, number of observed occurrences indicates if presence locations from the park were available for model development, and minimum distance to occurrence is the minimum distance from the park boundary to a known occurrence used in model development.

Park name	Potential suitable area (acres)	Percent of park (%)	Number of observed occurrences	Minimum distance to occurrence (km)
Joshua Tree National Park	506,459	64%	308	0
Death Valley National Park	668,933	20%	2	0
Tule Springs Fossil Beds National Monument	4,160	18%	0	5
Castle Mountains National Monument	3,520	17%	0	18
Mojave National Preserve	106,067	7%	0	11
Zion National Park	117	<1%	0	117
Arches National Park	38	<1%	0	289
Timpanogos Cave National Monument	0	0	0	353
Pipe Spring National Monument	0	0	0	120
Hovenweep National Monument	0	0	0	193
Manzanar National Historic Site	0	0	0	70
Bryce Canyon National Park	0	0	0	163
Great Basin National Park	0	0	0	187
Cedar Breaks National Monument	0	0	0	148

**Table 3:** Regional analysis table for bishop’s goutweed (*Aegopodium podagraria*) in Great Lakes EPMT unit including the potential suitable area for the one percentile threshold, percent of park is the percent of the park area that is classified as potentially suitable, number of observed occurrences indicates if presence locations from the park were available for model development, and minimum distance to occurrence is the minimum distance from the park boundary to a known occurrence used in model development.

Park name	Potential suitable area (acres)	Percent of park (%)	Number of observed occurrences	Minimum distance to occurrence
Keweenaw National Historical Park	1,826	98%	0	0
Ice Age National Scenic Trail	153	97%	0	9
Lower Saint Croix National Scenic Riverway	8,336	74%	9	0
Indiana Dunes National Lakeshore	11,393	72%	0	34
Mississippi National River and Recreation Area	38,322	71%	0	2
Saint Croix National Scenic Riverway	45,798	66%	0	1
Sleeping Bear Dunes National Lakeshore	44,427	63%	0	1
Pictured Rocks National Lakeshore	44,935	61%	3	0
Apostle Islands National Lakeshore	29,385	40%	1	0
Voyageurs National Park	23,201	11%	0	9
Isle Royale National Park	50,343	9%	0	42
Grand Portage National Monument	67	9%	0	21

### Local habitat suitability

When evaluating the local extent model ensemble predictions, Joshua Tree National Park contains a predicted 204,957 ha of fountain grass suitable habitat (64% of the park; calculated using the one percentile threshold and including any location where at least half the models agreed it was suitable). There are several known occurrence points, most of which are in the western portion of the park (Fig 2). Discussions with park staff revealed a new population found within the middle of the park occurred in predicted suitable habitat from the model ensemble. Portions of the remote eastern portion of the park, which has not been surveyed, contain suitable habitat as well, and managers discussed wanting to prioritize surveys in those locations after viewing the mapped predictions. Within Pictured Rocks National Lakeshore, there are an estimated 18,185 ha of suitable habitat (61% of the park). Two of the known occurrence points occur within the park boundary (Fig 2). Areas of high suitable habitat were predicted in the northeastern portion of the park in the coastal grassland and shrubland vegetation and along known trails and roads (Fig 2).

**Fig 4:**
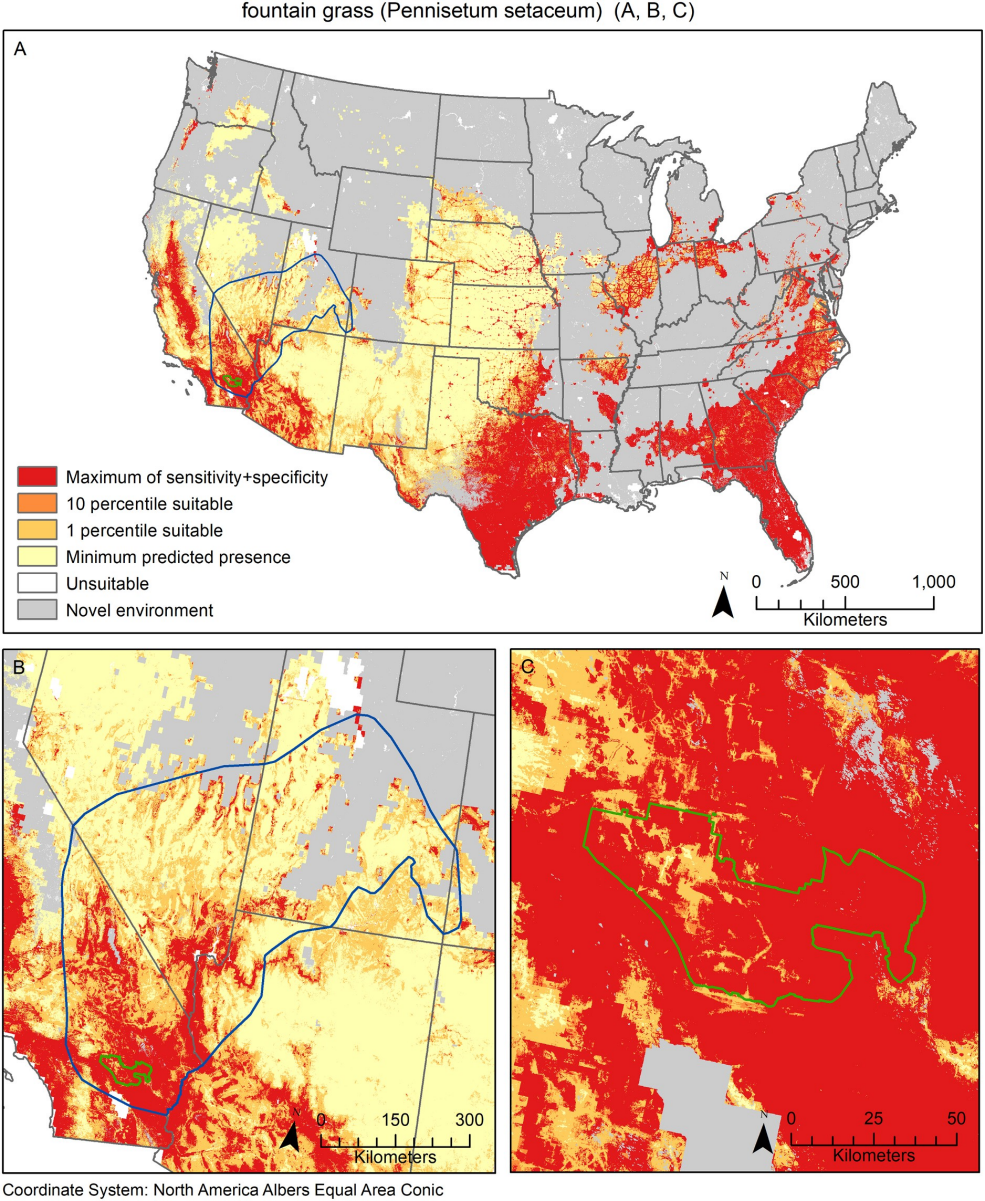
Fountain grass (*Pennisetum setaceum*) models of four different thresholds including minimum predicted presence (MPP), one percentile, ten percentile and maximum of sensitivity plus specificity (MSS). Model predictions are shown at three scales; A) national, B) regional (Lake Mead Exotic Plant Management Team region in blue) and C) local (Joshua Tree National Park in green).

The threshold values for both species provided a range of potential ways to view and interpret the results. By definition, the MPP provides the most inclusive predictions of suitable habitat while the ten percentile threshold is the most conservative and the differences between these can be substantial (Fig 4). Providing a spectrum of threshold values to view the results allows users to select an option that suits their management or decision making needs.

## Discussion

Our results suggest that this methodology provides a framework to balance efficient automation with species-specific model tuning to produce invasive species habitat suitability models useful for different management contexts and at multiple geographic extents. These models provide an entry point for an iterative modeling workflow. The methodology is built on readily available occurrence information and environmental variables covering the contiguous U.S. to provide efficiencies in developing large numbers of species-specific models. Using these inputs with the methodology provides a consistent modeling approach for multiple species that can be reviewed and compared. Similar frameworks for invasive species have been suggested however they are often conceptual, have limited human input or suggest the development of multiple models across spatial scales [24,58–61]. Sofaer et al. [12] identified the development of credible and repeatable models as an important issue limiting the use of species distribution models, and this framework provides a credible process as illustrated in the evaluation table (Table 4) and outputs such as response curves for expert review that are easily repeatable (Table B and Figure A in S1 File). This framework also supports iterative modeling, as input from users on environmental variables or new data can be readily incorporated to produce new model versions [12,59]. In addition, data quality is the fundamental driver of model quality which is why we gathered presence information from multiple sources and subjected them to rigorous, but generally automated, filtering [62,63]. As model outputs are shared with local practitioners, additional presence locations may become available for use in subsequent iterations. We considered alternative assumptions about the available environment related to invader spread (weighting points farther from others more [KDE] or basing weights solely on density of sampling [target]), corresponding to different sets of background locations [36,38]. Further, we used human input to evaluate the natural history of the species and select species-specific covariates, but for efficiency and scalability, we limited the selection to a set of variables designed to encompass and represent factors that limit plant distributions in different regions of the country.

**Table 4:** Model assessment rubric from Sofaer et al. [12] for fountain grass and goutweed models.

		Fountain grass	Goutweed
Species Data	Presence data quality	Acceptable: Location data evaluated for accuracy (taxonomic, spatial coordinates). Locations compared with reported distributions.	Acceptable: Location data evaluated for accuracy (taxonomic, spatial coordinates). Locations compared with reported distributions.
	Absence/ background data	Acceptable: Background data selected using target background approach to reflect sampling biases of invasive plants or weighted based on presence point density (mimic spreading species with less background at expanding edges).	Acceptable: Background data selected using target background approach to reflect sampling biases of invasive plants or weighted based on presence point density (mimic spreading species with less background at expanding edges).
	Evaluation data	Acceptable: Cross-validation of training data.	Acceptable: Cross-validation of training data.
Environmental Predictors	Ecological and predictive relevance	Acceptable: Predictors chosen based on natural history information for a perennial C4 grass.	Acceptable: Predictors chosen based on natural history information.
	Spatial and temporal alignment	Acceptable: Predictors match sampling period as closely as possible. Used available resolution closest to that desired for mapped products.	Acceptable: Predictors match sampling period as closely as possible. Used available resolution closest to that desired for mapped products.
Modeling Process	Algorithm choice	Acceptable: Used a range of algorithms (regression based, tree based, machine learning) that were evaluated separately based on *a priori* criteria for inclusion in final model.	Acceptable: Used a range of algorithms (regression based, tree based, machine learning) that were evaluated separately based on *a priori* criteria for inclusion in final model.
	Sensitivity	Acceptable: Evaluated five different algorithms and two background generation methods. Analyzed each algorithm’s settings separately based on *a priori* criteria.	Acceptable: Evaluated five different algorithms and two background generation methods. Analyzed each algorithm’s settings separately based on *a priori* criteria.
	Statistical rigor	Acceptable: Examined collinearity issues and visually evaluated residual map for spatial patterns.	Acceptable: Examined collinearity issues and visually evaluated residual map for spatial patterns.
	Performance	Acceptable: Evaluated multiple evaluation metrics to ensure they met *a priori* criteria. Visually examined mapped products to evaluate ecological plausibility. Included independent occurrence data set.	Acceptable: Evaluated multiple evaluation metrics to ensure they met *a priori* criteria. Visually examined mapped products to evaluate ecological plausibility.
	Model review	Acceptable: Review by regional species experts of initial response curves and regional and local maps experts pointed to independent data for evaluation.	Interpret with caution: Reviewed only by model developers. Needs regional species expert review.
Model Products	Mapped products	Acceptable: Ensemble of binary maps created for various thresholds that correspond to different intended uses.	Acceptable: Ensemble of binary maps created for various thresholds that correspond to different intended uses.
	Interpretation support products	Ideal: Model attributes described. Invasive plant management community engaged in development of models and the format of delivery.	Ideal: Model attributes described. Invasive plant management community engaged in development of models and the format of delivery.
	Reproducibility	Ideal: Inputs, scripts, settings, and results available.	Ideal: Inputs, scripts, settings, and results available.
	Iterative	Interpret with caution: First iteration	Interpret with caution: First iteration

Our results provide one model ensemble prediction for each species that can be interpreted across broad to local extents whereas most published single invasive species maps are only applicable to one or two extents (Table 1). At the national extent, these models show the current status or scope of the problem across the nation, highlighting regions of invasion or potential invasion that may be geographically distant from one another. For example, fountain grass is known to be a problematic invader in the west; however, the national model shows that the southeast portion of the country could also be susceptible to invasion (Fig 2). Interpreting the models at the national scale assesses the species’ impact across very different ecological regions of the country and the risk of invasion could be overlooked if the model was not developed at the national scale [5]. These models can also be interpreted regionally to assess the potential for invasion in the context of multiple land ownerships and uses. At the regional level, we identified multiple national parks that have not detected the invasive species of concern but likely have suitable habitat based on our model predictions (Tables 2 and 3). The use of the model at this regional extent can assess the level of risk of an invader by evaluating the potential suitable habitat for a management area as it relates to the proximity to the closest known occurrence based on the data used to develop the model [64]. Finally, at the local extent, these models can be used to guide early detection and rapid-response (EDRR) or control efforts. Model results can be viewed within administrative boundaries and at a resolution of 90 m, providing enough detail for managers to guide surveys to local incipient populations and to target control actions towards locations that will contribute to containment or impact reduction [65].

We developed our models with Sofaer et al.’s [12] model assessment rubric in mind and evaluated our models using this framework (Table 4). Our models consistently scored in the ‘acceptable practices’ category with a few metrics scoring as ‘ideal’ (‘interpretation support products’ and ‘reproducibility’) and two as ‘interpret with caution’ (‘model review’ for goutweed and ‘iterative’ for both; the latter is by design as we were interested in defining an entry point for iterative modeling). For many models, it would be impossible to score ‘ideal’ using readily available data rather than data collected based on a statistically designed study for the specific species. The ‘interpret with caution’ categories could move to ‘acceptable practices’ with a second iteration. As the model results are evaluated and used in the field, additional data will be provided and practitioners and experts will also have an opportunity to review and evaluate the model results. Further, our model results provide a ranking of the environmental variable contribution to the model combined with figures of the response curves (Table B and Figure A in S1 File). Compiling and presenting these results not only inform the modelers what factors may be important and how the species may respond to those factors but also serve as an important resource for practitioners and species experts to evaluate the model credibility. This can provide another aspect of model validation or enable model iteration and improvement.

As the practice of modeling habitat suitability evolves and expands, challenges arise with how much of the process can be automated versus which areas need human intervention. There is a desire to develop habitat suitability models for collections of species using similar methods to support a number of initiatives [58,60,66]. In the development of our framework, we included places in the model process for human input. We found the estimation, prediction, pre- and post-processing of models can be automated reasonably well. We built in automation of the occurrence data processing, model input data preprocessing (via SAHM) and model product development while having human intervention at key steps such as environmental variable selection for each species and examination of model parameters to determine overfitting and ecoplausibility (Fig 1). By using SAHM as opposed to keeping the scripting in R, we are able to take advantage of the well-developed graphical user interface for the human inputs of variable selection and examining model outputs (Fig 1). In addition, SAHM has a more efficient and robust method for model tracking and documentation [42]. We developed this approach with the understanding that human time and knowledge are valuable, whereas computing time and power are now readily available [42]. Humans provide judgment, adaptability, motivation, and can draw on experiences while automation can improve consistency, speed, and advanced computation [67]. This combination provides the speed and consistency of automation while incorporating the species-specific tailoring of human knowledge and critical junctures in the modeling process.

A key aspect in our approach is a framework for iterative modeling in which use of the model will improve future versions (‘Expert Review’ and ‘Model Application’ feed back into model development, Fig 1). Iterative modeling provides an opportunity to develop a suite of necessarily imprecise models for multiple species and improve models through their use. Models can undergo a new iteration upon the collection of new occurrence data, new environmental variables or from the review of the response curves and spatial results from experts. For example, as remote sensing technology improves spatial resolution and coverage, these new environmental data can be incorporated into the environmental variable library to provide additional predictive power that has been shown to effectively map invasive spices populations in localized examples [9]. As shown with gypsy moth, operational iterative modeling can improve models over time and lead to better informed management actions [15]. Our models represent the first iteration of these predictions. Future versions will need to be determined based on the frequency and quality of incoming data or expert review. Iterative monitoring and modeling are essential to continually improving models to inform decisions and management actions [4,59,68]. For example, model-informed decision-making that includes surveys for new populations can be used to assess model performance and the information gathered can be included in iterative model updates.

There are many pitfalls, assumptions, and caveats of correlative species distribution models [11] that are evaluated in Table 4. Despite these caveats, species distribution models can be useful in informing management activities (see examples in [12]). We addressed the primary challenges we identified as impacting distribution modeling. We incorporated multiple factors that may control species distribution at different scales, moving beyond strictly climate envelopes. This suite covers a range of categories of predictor type and included the best available resolutions for nationally consistent data. Since our scope was limited to plants, we created a suite of common distributional drivers that drive many invasive plant species’ distribution while also moving beyond readily available bioclimatic variables [41]. However, this suite could easily be augmented to include variables that would also be applicable to other taxonomic groups. We used aggregated occurrence data and accounted for potential biases in two different ways to overcome limitations in available data. Our framework allows for the flexibility to include other background selection approaches that have been recently proposed (e.g.,[69]) in this process if they demonstrate improvements to the modeling process. These products could be used to develop sampling schemes that could provide more information to improve models in the future [15,26]. Finally, we use model ensembles to encompass uncertainty among algorithms, and visualize response curves to assess overfitting and ecoplausibility and compare among models [8,36].

Our methodology and results present and demonstrate an approach to develop and use national-scale invasive species habitat suitability models that support multi-scale interpretation that can support multiple uses and audiences. We provide a framework that leverages the skills of modeling experts, the species-specific knowledge from field managers and researchers, and a process for iterative modeling. We use readily available occurrence and national extent environmental variable data to develop invasive species suitability models that are grounded in acceptable practices for species distribution modeling [11,36]. Future development should focus on application of the framework to other invasive plant species, implementation of the iterative process, and delivery of model results to practitioners. The model results are seamless across the continental U.S. and can easily be applied to other species of interest. Once the framework has been applied to a large set of species, the set of model results can be combined to identify potential hot spots of invasion or efficiencies in management across species (e.g., single crew targeting multiple species in an area). A key step is to provide a mechanism to easily transfer the results from this methodology to managers making decisions at these different geographic extents.

## Supporting information

S1 File Supplementary material that includes environmental variables considered for invasive habitat suitability modeling (Table A), the percent contribution of each environmental variable by model for fountain grass and goutweed (Table B), environmental response curves for fountain grass and goutweed final habitat suitability models (Figure A), and model performance metrics for fountain grass and goutweed (Table C). (DOCX)
