# Supplementary material for: A modeling workflow that balances automation and human intervention to inform invasive plant management decisions at multiple spatial scales
Source: PLoS One. 2020 Mar 9;15(3):e0229253. doi: 10.1371/journal.pone.0229253 (PMC7062246; doi:10.1371/journal.pone.0229253)
Supplement: S1 File — (DOCX) [file pone.0229253.s001.docx]

**SUPPLEMENTARY MATERIAL**

**Table A**: List of environmental variables considered for invasive habitat suitability modeling including their name, spatial (cell) resolution, unit, description and source.

| **Variable** | **Resolution** | **Units** | **Description** | **Source** |
| --- | --- | --- | --- | --- |
| Annual Mean Temperature (bio1)  1981 - 2010 | 800 m | °C | Annual mean temperature has climate inputs that consist of monthly mean maximum and minimum temperatures, which are averaged across the year to acquire annual mean temperature. The annual mean temperature approximates the total energy inputs for an ecosystem. | [PRISM](http://www.prism.oregonstate.edu) & O’Donnell and Ignizio 2012 ^1^ |
| Mean Diurnal Range (bio2)  1981 - 2010 | 800 m | °C | Mean diurnal range is the mean of all the averaged, monthly temperature extremes whose inputs are monthly mean maximum and minimum temperatures. It is calculated by finding the difference between the maximum and minimum temperature for each month, and then averaging these values, which can inform the relevance of temperature fluctuation to different species. | [PRISM](http://www.prism.oregonstate.edu) & O’Donnell and Ignizio 2012 ^1^ |
| Isothermality (bio3)  1981 - 2010 | 800 m | percent | Isothermality quantifies how much the day-to-night temperatures oscillate relative to the summer-to-winter oscillations. Inputs include monthly mean maximum and monthly mean minimum temperatures. | [PRISM](http://www.prism.oregonstate.edu) & O’Donnell and Ignizio 2012 ^1^ |
| Temperature Seasonality (std dev) (bio4)  1981 - 2010 | 800 m | stddev * 100 | Temperature seasonality is the amount of temperature variation over the given period based on the standard deviation of monthly temperature averages. This is calculated by averaging the minimum temperature and maximum temperature for each month and then calculating the standard deviation of the 12 mean monthly temperature values. | [PRISM](http://www.prism.oregonstate.edu) & O’Donnell and Ignizio 2012 ^1^ |
| Temperature Seasonality (std dev) (bio4a)  1981 - 2010 | 800 m | stddev * 100 | Temperature seasonality is the amount of temperature variation over the given period based on the standard deviation of monthly temperature averages. Values are first converted to degrees Kelvin so negative temperature values do not occur and avoids the possibility of having to divide by zero. This is calculated by averaging the minimum temperature and maximum temperature for each month and then calculating the standard deviation of the 12 mean monthly temperature values. | [PRISM](http://www.prism.oregonstate.edu) & O’Donnell and Ignizio 2012 ^1^ |
| Maximum Temperature Warmest Month (bio5)  1981 - 2010 | 800 m | °C | Maximum temperature of warmest month is the monthly mean maximum temperature occurrence over a given period. This can inform whether species distributions are affected by warm anomalies throughout the year. | [PRISM](http://www.prism.oregonstate.edu) & O’Donnell and Ignizio 2012 ^1^ |
| Minimum Temperature Coldest Month (bio6)  1981 - 2010 | 800 m | °C | Minimum temperature of coldest month is the monthly mean minimum temperature occurrence over a given period. This can inform whether species distributions are affected by cold anomalies throughout the year. | [PRISM](http://www.prism.oregonstate.edu) & O’Donnell and Ignizio 2012 ^1^ |
| Temperature Annual Range (bio7)  1981 - 2010 | 800 m | °C | Temperature annual range is a measure of temperature variation over a given period calculated by subtracting minimum temperature of coldest month (BIO 6) from maximum temperature of warmest month (BIO5). This can inform whether species distributions are affected by ranges of extreme climatic conditions. | [PRISM](http://www.prism.oregonstate.edu) & O’Donnell and Ignizio 2012 ^1^ |
| Mean Temperature of Wettest Quarter (bio8)  1981 - 2010 | 800 m | °C | Mean temperature of wettest quarter approximates mean temperatures that prevail during the wettest season. It is calculated by identifying the three consecutive months with the highest cumulative precipitation total; The maximum and minimum temperature for each of the 3 selected months is averaged, and the monthly averages are averaged. | [PRISM](http://www.prism.oregonstate.edu) & O’Donnell and Ignizio 2012 ^1^ |
| Mean Temperature of Driest Quarter (bio9)  1981 - 2010 | 800 m | °C | Mean temperature of driest quarter approximates mean temperatures that prevail during the driest season. It is calculated by identifying the three consecutive months with the lowest cumulative precipitation total; The maximum and minimum temperature for each of the 3 selected months is averaged, and the monthly averages are averaged. | [PRISM](http://www.prism.oregonstate.edu) & O’Donnell and Ignizio 2012 ^1^ |
| Mean Temperature of Warmest Quarter (bio10)  1981 - 2010 | 800 m | °C | Mean temperature of warmest quarter approximates mean temperatures that prevail during the warmest season. It is calculated by identifying the warmest quarter of the year (the average temperatures of each month in the quarter are summed). The maximum and minimum temperatures are averaged for each of the 3 selected months, and the monthly averages are averaged. | [PRISM](http://www.prism.oregonstate.edu) & O’Donnell and Ignizio 2012 ^1^ |
| Mean Temperature of Coldest Quarter (bio11)  1981 - 2010 | 800 m | °C | Mean temperature of coldest quarter approximates mean temperatures that prevail during the coldest season. It is calculated by identifying the coldest quarter of the year (the average temperatures of each month in the quarter are summed). The maximum and minimum temperatures are averaged for each of the 3 selected months, and the monthly averages are averaged. | [PRISM](http://www.prism.oregonstate.edu) & O’Donnell and Ignizio 2012 ^1^ |
| Annual Precip (bio12)  1981 - 2010 | 800 m | mm | Annual precipitation is the sum of all total monthly precipitation estimates. This approximates the total water inputs which can inform the importance of water availability to species distributions. | [PRISM](http://www.prism.oregonstate.edu) & O’Donnell and Ignizio 2012 ^1^ |
| Precipitation of Wettest Month (bio13)  1981 - 2010 | 800 m | mm | Precipitation of wettest month is the total precipitation that prevails during the wettest month. Extreme precipitation conditions during the year influences a species' potential range. If is derived from climate normals, then the wettest month is based on a span of years. | [PRISM](http://www.prism.oregonstate.edu) & O’Donnell and Ignizio 2012 ^1^ |
| Precipitation of Driest Month (bio14)  1981 - 2010 | 800 m | mm | Precipitation of driest month is the total precipitation that prevails during the driest month. Extreme precipitation conditions during the year influences a species' potential range. If is derived from climate normals, then the driest month is based on a span of years. | [PRISM](http://www.prism.oregonstate.edu) & O’Donnell and Ignizio 2012 ^1^ |
| Precipitation Seasonality (Cv) (bio15)  1981 - 2010 | 800 m | percent | Precipitation seasonality is a measure of the variation in monthly precipitation totals over the course of the year. This index is the ratio of the standard deviation of the monthly total precipitation to the mean monthly total precipitation (aka coefficient of variation) and is expressed as a percentage | [PRISM](http://www.prism.oregonstate.edu) & O’Donnell and Ignizio 2012 ^1^ |
| Precipitation of Wettest Quarter (bio16)  1981 - 2010 | 800 m | mm | Precipitation of wettest quarter approximates total precipitation that prevails during the wettest season. The three consecutive months with the highest cumulative precipitation total are identified, and then the precipitation values are summed for all three months. | [PRISM](http://www.prism.oregonstate.edu) & O’Donnell and Ignizio 2012 ^1^ |
| Precipitation of Driest Quarter (bio17)  1981 - 2010 | 800 m | mm | Precipitation of driest quarter approximates total precipitation that prevails during the driest season. The three consecutive months with the lowest cumulative precipitation total are identified, and then the precipitation values are summed for all three months. | [PRISM](http://www.prism.oregonstate.edu) & O’Donnell and Ignizio 2012 ^1^ |
| Precipitation of Warmest Quarter (bio18)  1981 - 2010 | 800 m | mm | Precipitation of warmest quarter approximates total precipitation that prevails during the warmest season. It is calculated by identifying the warmest quarter of the year (the average temperatures of each month in the quarter are summed; the quarter with the highest value is selected), and the precipitation values for the three months in this quarter are then summed. | [PRISM](http://www.prism.oregonstate.edu) & O’Donnell and Ignizio 2012 ^1^ |
| Precipitation of Coldest Quarter (bio19)  1981 - 2010 | 800 m | mm | Precipitation of coldest quarter approximates total precipitation that prevails during the coldest season. It is calculated by identifying the coldest quarter of the year (the average temperatures of each month in the quarter are summed; the quarter with the lowest value is selected), and the precipitation values for the three months in this quarter are then summed. | [PRISM](http://www.prism.oregonstate.edu) & O’Donnell and Ignizio 2012 ^1^ |
| Minimum Winter Temperature  1981 – 2010 | 4,000 m | °C | Minimum temperature of winter months (December through February) is the monthly mean minimum temperature over the given time period. | [Climate Engine](https://app.climateengine.org/)^2^ |
| Mean Spring Temperature  1981 – 2010 | 4,000 m | °C | Mean temperature of spring months (March through June) | [Climate Engine](https://app.climateengine.org/)^2^ |
| Maximum Mean Summer Temperature  1981 - 2010 | 4,000 m | °C | Maximum temperature of summer months (June through August) | [Climate Engine](https://app.climateengine.org/)^2^ |
| Maximum Summer Temperature  1981 - 2010 | 4,000 m | °C | Mean maximum temperature of summer months (June through August) | [Climate Engine](https://app.climateengine.org/)^2^ |
| Mean PET Fall  1981 - 2010 | 4,000 m | mm | Potential water deficit of fall months (October through November) calculated by averaging potential evapotranspiration subtracted from precipitation over a given time period. | [Climate Engine](https://app.climateengine.org/)^2^ |
| Mean PET Spring  1981 - 2010 | 4,000 m | mm | Potential water deficit of spring months (March through June) calculated by averaging potential evapotranspiration subtracted from precipitation over a given time period. | [Climate Engine](https://app.climateengine.org/)^2^ |
| Mean PET Summer  1981 - 2010 | 4,000 m | mm | Potential water deficit of summer months (June through August) calculated by averaging potential evapotranspiration subtracted from precipitation over a given time period. | [Climate Engine](https://app.climateengine.org/)^2^ |
| Mean PET Spring  1981 - 2010 | 4,000 m | mm | Potential water deficit of early spring months (March through May) calculated by averaging potential evapotranspiration subtracted from precipitation over a given time period. | [Climate Engine](https://app.climateengine.org/)^2^ |
| Mean PET  1981 - 2010 | 4,000 m | mm | Potential water deficit of fall to spring months (October to June) calculated by averaging potential evapotranspiration subtracted from precipitation over a given time period. | [Climate Engine](https://app.climateengine.org/)^2^ |
| Mean Precipitation Spring  1981 - 2010 | 4,000 m | mm | Mean precipitation of early spring (March-May) | [Climate Engine](https://app.climateengine.org/)^2^ |
| Mean Precipitation Spring  1981 - 2010 | 4,000 m | mm | Mean precipitation of spring months (March through June) | [Climate Engine](https://app.climateengine.org/)^2^ |
| Mean Precipitation March  1981 - 2010 | 4,000 m | mm | Mean precipitation of March | [Climate Engine](https://app.climateengine.org/)^2^ |
| Mean Precipitation March/Mean Precipitation Spring  1981 - 2010 | 4,000 m | fraction/mm | Mean precipitation of March divided by Mean precipitation of spring months (March through June) | [Climate Engine](https://app.climateengine.org/)^2^ |
| Mean Precipitation Growing Season  1981 - 2010 | 4,000 m | mm | Mean precipitation of the growing season (April through October) | [Climate Engine](https://app.climateengine.org/)^2^ |
| Mean Annual Precipitation  1981 - 2010 | 4,000 m | mm | Mean annual precipitation | [Climate Engine](https://app.climateengine.org/)^2^ |
| Evapotranspiration: Oct-Nov  2003 - 2017 | 1,000 m | mm | Mean monthly evapotranspiration of fall months (October through November) | [Climate Engine](https://app.climateengine.org/)^2^ |
| Evapotranspiration: Mar-Jun  2003 - 2017 | 1,000 m | mm | Mean monthly evapotranspiration of spring months (March through June) | [Climate Engine](https://app.climateengine.org/)^2^ |
| Evapotranspiration: Apr-Oct  2003 - 2017 | 1,000 m | mm | Mean monthly evapotranspiration of the growing season (April through October) | [Climate Engine](https://app.climateengine.org/)^2^ |
| Evapotranspiration: Oct-Jun  2003 - 2017 | 1,000 m | mm | Mean monthly evapotranspiration of fall to spring (October through June) | [Climate Engine](https://app.climateengine.org/)^2^ |
| Evapotranspiration: Mar-May  2003 - 2017 | 1,000 m | mm | Mean monthly evapotranspiration of spring months (March through May) | [Climate Engine](https://app.climateengine.org/)^2^ |
| Evapotranspiration: Jun-Aug  2003 - 2017 | 1,000 m | mm | Mean monthly evapotranspiration of summer months (June through August) | [Climate Engine](https://app.climateengine.org/)^2^ |
| Burning Index  1981 - 2010 | 4,000 m | 0-100 | Burning index mean (based off of the National Fire Danger Rating System) | [Climate Engine](https://app.climateengine.org/)^2^ |
| Landscape Condition Model  2017 | 90 m | 0-1 | The landscape condition model identifies human land uses (built infrastructure, agriculture, vegetation alteration, etc.) | [Nature Serve](http://www.natureserve.org/conservation-tools/modeling-landscape-condition)^3^ |
| Human Influence Index  1995 - 2004 | 1,000 m | 0-64 | Human influence index identifies anthropogenic impacts on the environment. It incorporates nine global data layers covering human population pressure (population density), human land use and infrastructure (built-up areas, nighttime lights, land use/land cover), and human access (coastlines, roads, railroads, navigable rivers). | [NASA - SEDAC](http://sedac.ciesin.columbia.edu/data/set/wildareas-v2-human-influence-index-geographic)^4^ |
| Percent Clay (0-5cm) | 100 m | percent | Mean percent clay cover in the first 0-5cm of the soil horizon | [POLARIS](https://www.sciencedirect.com/science/article/pii/S0016706116301434)^5^ |
| Percent Sand (0-5cm) | 100 m | percent | Mean percent sand cover in the first 0-5cm of the soil horizon | [POLARIS](https://www.sciencedirect.com/science/article/pii/S0016706116301434)^5^ |
| Percent Calcium Carbonate in Soil | 100 m | percent | Mean percent calcium carbonate in the first 0-5cm of the soil horizon | [POLARIS](https://www.sciencedirect.com/science/article/pii/S0016706116301434)^5^ |
| Pore Size Distribution Index | 100 m | none | Mean pore size distribution index in the first 0-5cm of the soil horizon | [POLARIS](https://www.sciencedirect.com/science/article/pii/S0016706116301434)^5^ |
| Available Water Content (depth, cm) | 100 m | m3/m2 | Variance of available water content in the first 0-5cm of the soil horizon | [POLARIS](https://www.sciencedirect.com/science/article/pii/S0016706116301434)^5^ |
| Available Water Content (depth, cm) | 100 m | m3/m3 | Mean available water content in the first 0-5cm of the soil horizon | [POLARIS](https://www.sciencedirect.com/science/article/pii/S0016706116301434)^5^ |
| Soil Water Content at Field Capacity (0-5 cm) | 100 m | m3/m3 | Mean soil water content at field capacity in the first 0-5cm of the soil horizon | [POLARIS](https://www.sciencedirect.com/science/article/pii/S0016706116301434)^5^ |
| Depth to Restriction Layer | 100 m | cm | Mean depth to restriction layer | [POLARIS](https://www.sciencedirect.com/science/article/pii/S0016706116301434)^5^ |
| Remoteness (Night Lights) | 250 m | bright-  ness | Nighttime lights of 2011. Cloud-free composites use visible and infrared sensors to capture imagery. | [NOAA](https://ngdc.noaa.gov/eog/dmsp/downloadV4composites.html) |
| Land Surface Temperature (Modis) | 1000 m | 0-254 | Land surface temperature/emissivity | [NASA](https://modis-land.gsfc.nasa.gov/temp.html) |
| Maximum Burn Severity  1984 - 2015 | 30 m | 0 to 6 | Maximum burn severity | [MTBS](https://www.mtbs.gov/product-descriptions)^6^ |
| Burn Frequency  1984 - 2015 | 30 m | 0 to 15 | Burn frequency | [MTBS](https://www.mtbs.gov/product-descriptions)^6^ |
| Year of Last Fire | 30 m | 1 to 32 | Year of the last fire from 1984 to 2015 | [MTBS](https://www.mtbs.gov/product-descriptions)^6^ |
| Severity of Last Fire  1984 - 2015 | 30 m | 0 to 6 | Burn severity of last fire | [MTBS](https://www.mtbs.gov/product-descriptions)^6^ |
| Bare Ground Standard Deviation  2010 - 2016 | 250 m | percent stddev | Standard deviation of percent cover of bare ground over the given time period | [NASA - LP DAAC](https://lpdaac.usgs.gov/products/mod44bv006/)^7^ |
| Mean Tree Cover  2010 - 2016 | 250 m | percent | Mean tree cover over the given time period | [NASA - LP DAAC](https://lpdaac.usgs.gov/products/mod44bv006/)^7^ |

^1^ Daly, C., Halbleib, M., Smith, J.I., Gibson, W.P., Doggett, M.K., Taylor, G.H., Curtis, J., Pasteris, P.P. “Physiographically sensitive mapping of climatological temperature and precipitation across the conterminous United States”. International Journal of Climatology. (2008) 28, 2031–2064. <https://doi.org/10.1002/joc.1688>. AND

O’Donnell, M.S. and Ignizio, D.A. (2012). Bioclimatic predictors for supporting ecological applications in the conterminous United States. US Geological Survey Data Series, 691(10).

^2^Abatzoglou J. T. " Development of gridded surface meteorological data for ecological applications and modelling " International Journal of Climatology. (2011) doi: 10.1002/joc.3413.

^3^Hak, J.C. and P.J. Comer. 2017. Modeling Landscape Condition for Biodiversity Assessment – Application in Temperate North America. Ecological Indicators 82:206-216.

^4^Wildlife Conservation Society - WCS, and Center for International Earth Science Information Network - CIESIN - Columbia University. (2005). Last of the Wild Project, Version 2, 2005 (LWP-2): Global Human Influence Index (HII) Dataset (Geographic). Palisades, NY: NASA Socioeconomic Data and Applications Center (SEDAC). https://doi.org/10.7927/H4BP00QC. Accessed DAY MONTH YEAR.

^5^Chaney NW, Wood EF, McBratney AB, Hempel JW, Nauman TW, Brungard CW, Odgers NP. 2016. POLARIS: A 30-meter probabilistic soil series map of the contiguous United States. Geoderma 274: 54-67.

^6^Eidenshink, J., B. Schwind, K. Brewer, Z. Zhu, B. Quayle, and S. Howard. (2007). A project for monitoring trends in burn severity. Fire Ecology 3(1): 3-21.

^7^DiMiceli, C. M., Carroll, M. L., Sohlberg, R. A., Huang, C., Hansen, M. C., & Townshend, J. R. G. (2011). Annual global automated MODIS vegetation continuous fields (MOD44B) at 250 m spatial resolution for data years beginning day 65, 2000– 2010, collection 5 percent tree cover. College Park, MD: University of Maryland.

**Table B**: Percent contribution of each environmental variable by model for a) fountain grass and b) goutweed. Models included five algorithms (Boosted Regression Trees (BRT), generalized linear models (GLM), Multivariate adaptive regression splines (MARS), Maxent and Random forests (RF)) with two background sample approaches (target guild (target) and kernel density estimation (KDE)). The top three environmental variables for each model are highlighted.

a)

| Environmental variable | BRT KDE | BRT target | GLM KDE | GLM target | MARS KDE | MARS target | Maxent KDE | Maxent target | RF KDE | RF target |
| --- | --- | --- | --- | --- | --- | --- | --- | --- | --- | --- |
| Landscape Condition Model | 4 | 2 | NA | 3 | NA | 5 | 2 | 3 | 2 | 3 |
| Available Water Content, Mean (depth, cm) | 2 | NA | 7 | NA | 2 | NA | 1 | NA | 1 | NA |
| Available Water Content, Variance (depth, cm) | NA | NA | NA | 0 | NA | NA | 0 | 0 | 0 | 0 |
| Bare Ground Standard Deviation | NA | NA | NA | 0 | 0 | NA | 1 | 0 | 1 | 1 |
| Precipitation Seasonality (Cv) (bio15) | 18 | 6 | 5 | 5 | 6 | 4 | 8 | 3 | 14 | 5 |
| Percent Calcium Carbonate in Soil | 1 | NA | NA | NA | NA | NA | 0 | NA | 1 | NA |
| Percent Clay (0-5cm) | NA | 3 | NA | 17 | NA | 2 | NA | 2 | NA | 2 |
| Evapotranspiration: Oct-Jun | 17 | 5 | 27 | 4 | 25 | 7 | 16 | 3 | 20 | 4 |
| Human Influence Index | 19 | 8 | 21 | 7 | 27 | 9 | 21 | 9 | 36 | 7 |
| Land Surface Temperature (Modis) | 1 | NA | NA | NA | NA | NA | 1 | NA | 1 | NA |
| Burn Frequency | NA | NA | NA | NA | 0 | NA | 0 | 0 | 0 | 0 |
| Mean Precipitation Growing Season: Apr-Oct | 6 | 4 | NA | 10 | 8 | 14 | 7 | 1 | 2 | 2 |
| Mean PET Spring: Mar-Jun | 8 | 44 | NA | 12 | NA | 19 | 10 | 40 | 1 | 47 |
| Depth to Restriction Layer | 3 | 5 | 13 | 6 | 11 | 8 | 9 | 4 | 3 | 3 |
| Minimum Winter Temperature | 18 | 21 | 18 | 27 | 13 | 20 | 21 | 28 | 13 | 21 |
| Mean Tree Cover | 3 | 3 | 8 | 8 | 8 | 12 | 3 | 7 | 2 | 3 |

b)

| predictor | BRT KDE | BRT target | GLM KDE | GLM target | MARS KDE | MARS target | Maxent KDE | Maxent target | RF KDE | RF target |
| --- | --- | --- | --- | --- | --- | --- | --- | --- | --- | --- |
| Landscape Condition Model | **54** | NA | **34** | 2 | **38** | 2 | **47** | 4 | **44** | 2 |
| Available Water Content, Mean (depth, cm) | NA | 7 | NA | 13 | NA | 2 | NA | 1 | NA | 7 |
| Available Water Content, Variance (depth, cm) | 1 | NA | NA | NA | 3 | NA | 4 | NA | 4 | NA |
| Bare Ground Standard Deviation | 1 | NA | 2 | 1 | 2 | 1 | 1 | 0 | 2 | 3 |
| Mean Diurnal Range (bio2) | NA | NA | NA | NA | 3 | NA | 0 | 0 | 2 | 4 |
| Isothermality (bio3) | NA | NA | 5 | NA | 0 | NA | 1 | NA | 3 | NA |
| Percent Calcium Carbonate in Soil | NA | NA | NA | **16** | NA | 2 | 0 | 2 | 1 | 3 |
| Percent Clay (0-5cm) | 3 | 14 | NA | 2 | 0 | 0 | 1 | 3 | 4 | **13** |
| Evapotranspiration: Apr-Oct | **6** | NA | 9 | NA | 6 | 0 | 6 | 1 | 5 | 4 |
| Evapotranspiration: Oct-Nov | NA | NA | 1 | NA | 0 | 0 | 1 | 2 | 2 | 3 |
| Human Influence Index | 5 | NA | 7 | NA | 2 | NA | 4 | NA | 2 | NA |
| Mean PET Summer: Jun-Aug | **14** | 12 | 6 | **21** | 8 | **7** | 4 | **8** | 5 | **15** |
| Mean Annual Precipitation | 4 | **27** | NA | NA | 3 | **41** | 3 | **36** | 4 | **13** |
| Remoteness (Night Lights) | NA | NA | 1 | NA | 2 | 0 | 2 | 0 | 2 | 3 |
| Depth to Restriction Layer | 4 | NA | **11** | NA | **11** | 0 | 6 | 3 | 3 | 4 |
| Maximum Mean Summer Temperature | 5 | **17** | **15** | NA | **16** | 4 | 6 | 8 | **11** | 11 |
| Minimum Winter Temperature | 4 | **23** | NA | **40** | 0 | **41** | **7** | **28** | 4 | 12 |
| Mean Tree Cover | NA | NA | 8 | 5 | 5 | 0 | 6 | 2 | 2 | 3 |

a)


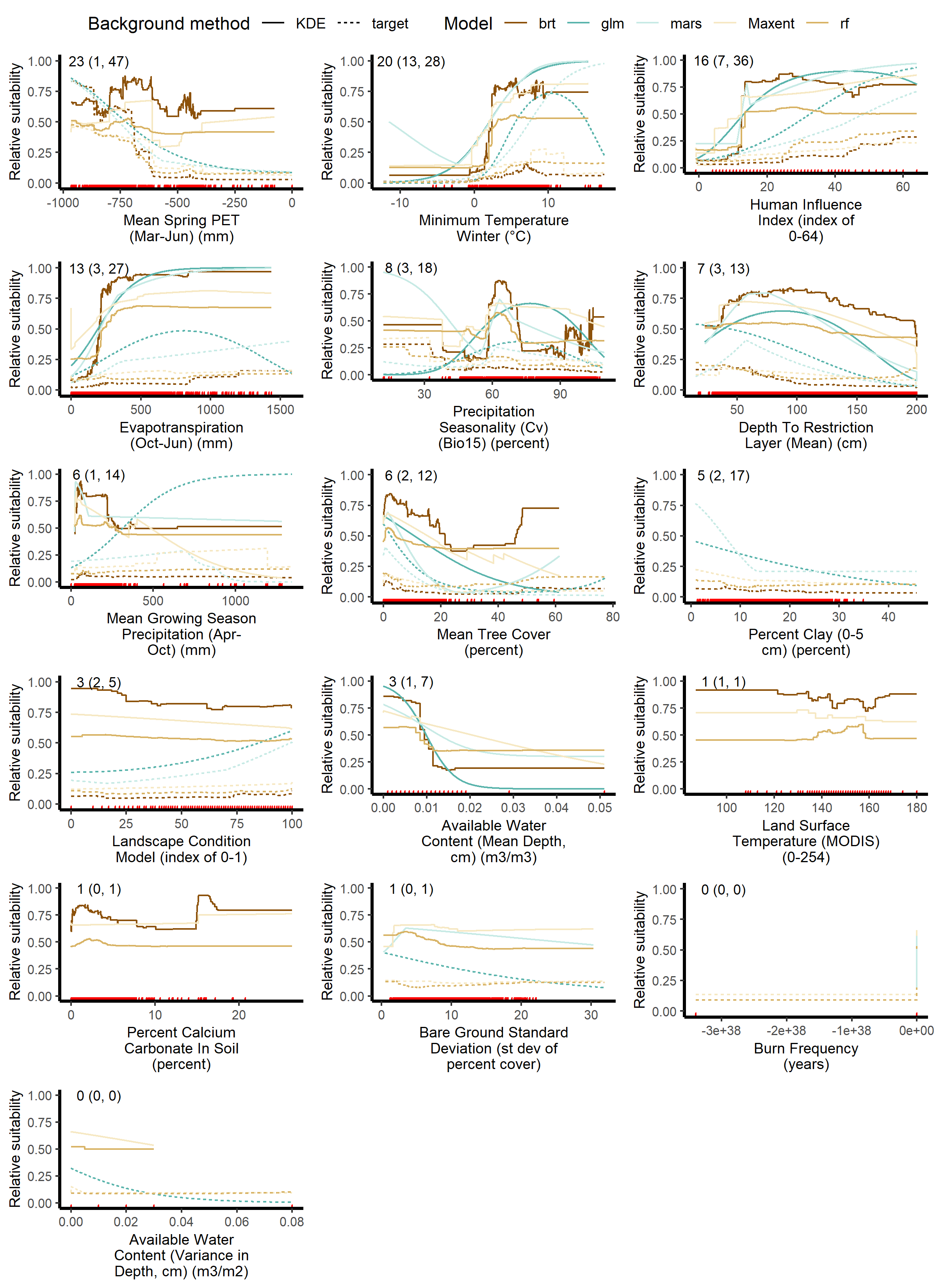


b)


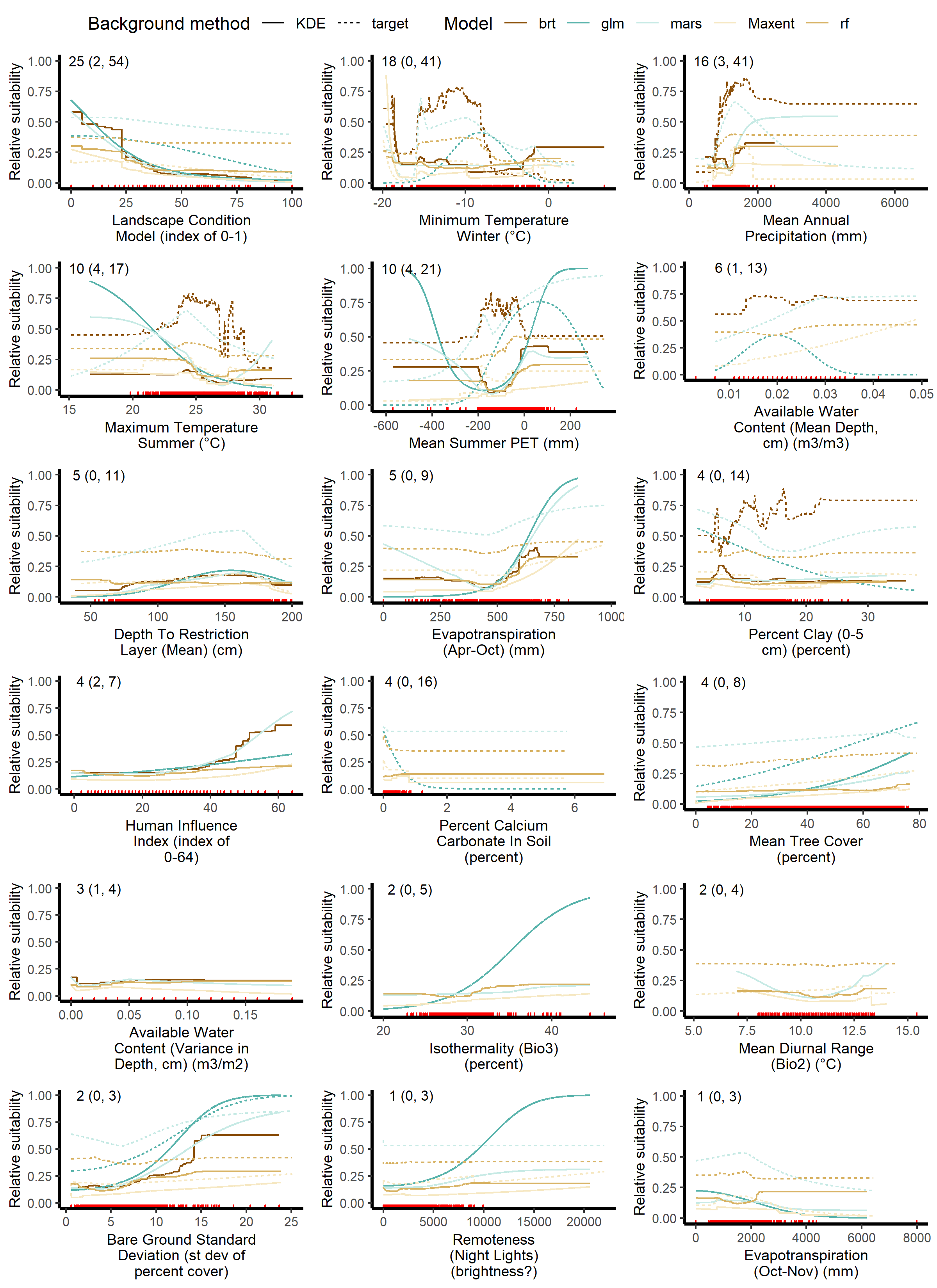


**Figure A**: Environmental response curves, ordered by average relative importance, for a) fountain grass and b) goutweed showing relative habitat suitability (y-axis) across the range of values in the occurrence data for each variable (x-axis). Each line represents one model algorithm and background point generation method for a total of 10 lines possible; missing lines indicate the predictor was dropped from that model. The numbers in the top left of each graph represent the average relative importance of the predictor with the range across runs model (all algorithm/ background method combinations) in parentheses. The graphs are arranged by relative importance, with the top left contributing most to models on average. The red lines along the x-axis represent presence points with those values.

**Table C**: Model performance metrics for a) fountain grass and b) goutweed including Area Under the receiver operating Curve (AUC), Area Under the Precision Recall Curve (AUC-PR), correlation coefficient (between observed and predicted), the sensitivity = specificity threshold used to calculate the threshold dependent metrics including percent correctly classified, sensitivity, specificity, kappa, true skill statistic. The threshold values for minimum predicted presence (MPP), the one percentile, ten percentile and maximum of sensitivity plus specificity values used to create suitability maps for both background location generation methods (kernel density estimator [KDE] and target background [target]). Model algorithms included boosted regression trees (BRT), generalized linear model (GLM), multivariate adaptive regression splines (MARS), Maxent and random forests (RF).

a)

| Model | **BRT** | | **GLM** | | **MARS** | | **Maxent** | | **RF** | |
| --- | --- | --- | --- | --- | --- | --- | --- | --- | --- | --- |
| Background | *KDE* | *target* | *KDE* | *target* | *KDE* | *target* | *KDE* | *target* | *KDE* | *target* |
| AUC | 0.981 (0.999) | 0.961 (0.985) | 0.923 (0.922) | 0.924 (0.935) | 0.936 (0.938) | 0.917 (0.920) | 0.955 (0.961) | 0.933 (0.940) | 0.985 (0.986) | 0.967 (0.966) |
| AUC-PR | 0.952 (0.998) | 0.915 (0.962) | 0.794 (0.791) | 0.832 (0.853) | 0.827 (0.827) | 0.819 (0.819) | 0.858 (0.876) | 0.85 (0.860) | 0.963 (0.963) | 0.928 (0.924) |
| Correlation Coefficient | 0.873 (0.956) | 0.809 (0.879) | 0.693 (0.691) | 0.705 (0.73) | 0.732 (0.735) | 0.705 (0.708) | 0.77 (0.783) | 0.738 (0.750) | 0.883 (0.885) | 0.825 (0.823) |
| Sensitivity = Specificity threshold | 0.618 (0.65) | 0.514 (0.5) | 0.568 (0.57) | 0.507 (0.49) | 0.464 (0.46) | 0.426 (0.43) | 0.385 (0.39) | 0.231 (0.24 ) | 0.645 (0.47) | 0.65 (0.4) |
| Percent correctly classified | 94.072 (99.014) | 90.429 (94.135) | 85.266 (85.248) | 84.503 (85.318) | 85.952 (86.119) | 83.687 (84.214) | 89.206 (90.206) | 86.081 (86.867) | 94.691 (94.24) | 91.738 (89.648) |
| Sensitivity | 0.908 (0.99) | 0.856 (0.941) | 0.853 (0.851) | 0.846 (0.858) | 0.858 (0.859) | 0.837 (0.843) | 0.882 (0.899) | 0.854 (0.862) | 0.878 (0.944) | 0.799 (0.899) |
| Specificity | 0.952 (0.990) | 0.922 (0.941) | 0.853 (0.853) | 0.845 (0.851) | 0.86 (0.862) | 0.837 (0.842) | 0.896 (0.903) | 0.863 (0.871) | 0.971 (0.942) | 0.962 (0.896) |
| Kappa | 0.849 (0.975) | 0.764 (0.857) | 0.649 (0.648) | 0.64 (0.658) | 0.664 (0.667) | 0.622 (0.633) | 0.736 (0.76) | 0.672 (0.69) | 0.861 (0.856) | 0.786 (0.753) |
| True Skill Statistic | 0.860 (0.980) | 0.779 (0.882) | 0.705 (0.704) | 0.691 (0.709) | 0.718 (0.721) | 0.674 (0.684) | 0.778 (0.802) | 0.717 (0.733) | 0.85 (0.886) | 0.761 (0.794) |
| Minimum predicted presence (MPP) threshold | 0.257 | 0.016 | 0 | 0 | 0.0004 | 0.001 | 0.021 | 0.001 | 0.596 | 0.533 |
| 1st percentile threshold | 0.65 | 0.217 | 0.089 | 0.079 | 0.062 | 0.064 | 0.085 | 0.073 | 0.689 | 0.652 |
| 10th percentile threshold | 0.881 | 0.627 | 0.445 | 0.422 | 0.371 | 0.339 | 0.276 | 0.207 | 0.85 | 0.767 |
| Maximum of sensitivity plus specificity (MSS) threshold | 0.66 | 0.48 | 0.55 | 0.48 | 0.35 | 0.45 | 0.66 | 0.66 | 0.66 | 0.66 |

b)

| Model | **BRT** | | **GLM** | | **MARS** | | **Maxent** | | **RF** | |
| --- | --- | --- | --- | --- | --- | --- | --- | --- | --- | --- |
| Background | *KDE* | *target* | *KDE* | *target* | *KDE* | *target* | *KDE* | *target* | *KDE* | *target* |
| AUC | 0.902 (0.916) | 0.951 (0.992) | 0.895 (0.908) | 0.917 (0.925) | 0.886 (0.906) | 0.923 (0.933) | 0.917 (0.940) | 0.939 (0.955) | 0.943 (0.944) | 0.953 (0.956) |
| AUC-PR | 0.576 (0.609) | 0.747 (0.925) | 0.482 (0.486) | 0.548 (0.555) | 0.43 (0.48) | 0.592 (0.623) | 0.622 (0.697) | 0.684 (0.727) | 0.741 (0.744) | 0.776 (0.783) |
| Correlation Coefficient | 0.529 (0.552) | 0.636 (0.761) | 0.46 (0.472) | 0.52 (0.525) | 0.437 (0.465) | 0.535 (0.549) | 0.587 (0.633) | 0.667 (0.695) | 0.609 (0.614) | 0.651 (0.657) |
| Sensitivity = Specificity threshold | 0.514 (0.51) | 0.749 (0.7) | 0.51 (0.52) | 0.51 (0.51) | 0.547 (0.56) | 0.489 (0.47) | 0.257 (0.25) | 0.183 (0.18) | 0.701 (0.46) | 0.693 (0.43) |
| Percent correctly classified | 83.479 (84.197) | 94.343 (95.457) | 82.997 (83.211) | 86.200 (86.254) | 82.516 (84.046) | 86.027 (86.202) | 86.386 (87.221) | 88.127 (88.493) | 94.622 (88.108) | 95.076 (89.213) |
| Sensitivity | 0.807 (0.842) | 0.805 (0.956) | 0.807 (0.832) | 0.862 (0.866) | 0.815 (0.839) | 0.847 (0.862) | 0.824 (0.874) | 0.848 (0.875) | 0.695 (0.883) | 0.784 (0.894) |
| Specificity | 0.838 (0.842) | 0.955 (0.954) | 0.832 (0.832) | 0.862 (0.862) | 0.826 (0.841) | 0.861 (0.862) | 0.867 (0.872) | 0.884 (0.886) | 0.967 (0.881) | 0.965 (0.892) |
| Kappa | 0.441 (0.466) | 0.663 (0.744) | 0.345 (0.357) | 0.433 (0.434) | 0.341 (0.374) | 0.425 (0.432) | 0.416 (0.45) | 0.473 (0.49) | 0.632 (0.474) | 0.688 (0.514) |
| True Skill Statistic | 0.646 (0.684) | 0.761 (0.910) | 0.639 (0.664) | 0.724 (0.728) | 0.641 (0.68) | 0.708 (0.724) | 0.691 (0.746) | 0.732 (0.761) | 0.662 (0.764) | 0.749 (0.786) |
| Minimum predicted presence (MPP) threshold | 0.01 | 0.297 | 0.0004 | 0.01 | 0.007 | 0 | 0.017 | 0.006 | 0.642 | 0.628 |
| 1st percentile threshold | 0.117 | 0.542 | 0.039 | 0.057 | 0.033 | 0.062 | 0.035 | 0.025 | 0.679 | 0.663 |
| 10th percentile threshold | 0.392 | 0.812 | 0.398 | 0.405 | 0.38 | 0.37 | 0.21 | 0.155 | 0.786 | 0.784 |
| Maximum of sensitivity plus specificity (MSS) threshold | 0.48 | 0.64 | 0.54 | 0.53 | 0.5 | 0.54 | 0.3 | 0.23 | 0.51 | 0.5 |
